# Dysregulated epidermal growth factor and tumor growth factor-beta receptor signaling through *GFAP-ACTA2* protein interaction in liver fibrosis

**DOI:** 10.12669/pjms.36.4.1845

**Published:** 2020

**Authors:** Sobia Hassan, Zil-e-Rubab, Hussain Shah, Summayya Shawana

**Affiliations:** 1 Dr. Sobia Hassan, MBBS, M. Phil. Altamash Institute of Dental Medicine, Karachi, Pakistan; 2 Dr. Zil-e-Rubab, MBBS, M. Phil, PhD. Ziauddin University Clifton Campus, Karachi, Pakistan; 3 Mr. Hussain Shah, Department of Chemical & Biomolecular Engineering, University of Melbourne, Australia; 4 Dr. Summayya Shawana, MBBs, M. Phil. Bahria University Medical & Dental College, Karachi, Pakistan

**Keywords:** *ACTA2*, *GFAP*, Liver fibrosis, Signalling by *EGFR*, *TGF*-beta receptor Signalling

## Abstract

**Objective:**

Viral hepatitis is associated with high morbidity and mortality. Identification of biological pathways involved in hepatic fibrosis resulting from chronic hepatitis C are essential for better management of patients. Constructing the *HCV*-human protein interaction network through bioinformatics may enable us to discover diagnostic biological pathways. We investigated to identify dysregulated pathways and gene enrichment based on actin alpha 2 (*ACTA2)* and glial fibrillar acidic protein (*GFAP)* interaction network analysis in hepatic fibrosis.

**Methods:**

This is an in-silico study conducted at Ziauddin University from March,2019 to September 2019. Enrichment and protein–protein interaction (PPI) network analysis of the identified proteins: *GFAP* and *ACTA2* along with their mapped gene data sets was performed using FunRich version 3.1.3.

**Results:**

Biological pathway grouping showed enrichment of proteins (85.7%) in signalling pathway by epidermal growth factor receptor (*EGFR*) and Tumor growth factor (*TGF)*-beta Receptor followed by signaling by *PDGF, FGFR* and *NGF* (71.4%) (p < 0.001). *SRC, PRKACA, PRKCA and PRKCD* were enriched in both *EGFR* and *TGF*-beta Signalling pathways.

**Conclusion:**

*EGFR* and *TGF-beta* signalling pathways were enriched in liver fibrosis. *SRC, PRKACA, PRKCA and PRKCD* were enriched and differentially expressed in both *EGFR* and *TGF*-beta signalling pathways

## INTRODUCTION

Hepatic fibrosis is the basic damage resulting from chronic hepatitis C (CHC) which is one of the prime health challenges.^1^ The ultramicroscopic changes occurring in hepatic fibrosis include activation of hepatic stellate cells (HSCs) which is triggered by injury to hepatocytes.^2^ The excessive secretion of collagen by activated HSCs induces hyperplasia and deposition of extracellular matrix (ECM), which ultimately leads to liver fibrosis and cirrhosis.^3^,^4^ When HSCs trans differentiate into proliferative, and contractile myofibroblasts, they express certain mesenchymal markers like alpha smooth muscle actin, encoded by Actin alpha 2- *ACTA2* gene which is an isoform of the vascular smooth muscle actin and is expressed in all stages and grades of CHC.^5^ The expression of contractile filaments, Alpha smooth muscle actin (*α SMA)*, was identified in stellate cells forming a basis of using smooth muscle actin as an immunohistochemical marker, which detects HSCs activation and a useful marker for early diagnosis of hepatic fibrosis.^6^ In addition to *ACTA2*, studies have shown that there is augmented expression of Glial Fibrillary Acidic -*GFAP*-positive HSCs in early stages of hepatic fibrosis.^7^ The *GFAP* gene encodes a class III intermediate filament protein expressed specifically in astrocytes of the central nervous system and their transformation capacity is well conserved.^8^ A study in rodents reported the expression of *GFAP* with an increased expression in the acute response to injury in the rat, and a decreased in the chronic one.^9^ It is reported that *GFAP* could represent a more useful marker than Alpha smooth muscle actin (*α-SMA*) of early HSCs activation and may be an early indicator of hepatic fibrogenesis. Our study done in 2014 revealed strong association of *GFAP* with the gold standard immunohistochemical marker, *ACTA2* suggesting that *GFAP* could be a useful indicator of early HSCs activation in CHC patients.^10^ The *GFAP* positive hepatic cells may be antecedents of the HSCs detected by *ACTA2* or they may denote a diverse subpopulation.^11^,^12^

Most common cause of hepatocellular carcinoma (HCC) in our country is viral hepatitis.^13^ It is vital that degree of cirrhosis is established by the clinician and risk factors for HCC are identified. Bioinformatics has enabled us to discover diagnostic biomarkers and to plan treatment modalities.^14^ In light of above facts, the purpose of this study is to identify dysregulated pathways and gene enrichment based on *ACTA2* and *GFAP* interaction network analysis in hepatic fibrosis.

## METHODS

This is an in-silico study. GFAP and ACTA2 were obtained by immunohistochemistry in previous study^9^ by one of the authors which was approved by the Ethical Review Committee (Ref. Code: 1601119ZRBIO) of Ziauddin University. The study was done from March-September 2019.

In this study, the gene expression and interaction of *GFAP* and *ACTA2* were analysed in silico. Immunoexpression of *GFAP* revealed substantial association with *ACTA2 (α-SMA*) in previous study concluding inverse relationship of *GFAP* with progression of fibrosis. Hence, *GFAP* could be characterized as useful marker for early hepatic stellate cells activation.

### Bioinformatics analysis

Enrichment and protein–protein interaction (PPI) network analysis of the identified proteins: *GFAP* and *ACTA2* along with their mapped gene data sets was performed using

### FunRich

*Functional Enrichment analysis tool* version 3.1.3 released on March 2017 http://www.funrich.org^14^ The enriched and depleted proteins were identified by calculating fold change for biological pathways, protein domains and site of expressions.

### Interaction network analysis

In FunRich software hypergeometric test, BH and Bonferroni test were applied. Normal and Overrepresented and gene ontology (GO) functional categories, significant interactions and pathways associated with datasets were identified by using the hypergeometric test and p-value correction with the BH and Bonferroni tests. Statistical cut-off of enrichment analyses was kept as default with a p=value <0.05 after Bonferroni correction.

## RESULTS

### Protein-Protein Interaction (PPI) Analysis of GFAP and ACTA2

**Fig.1:**
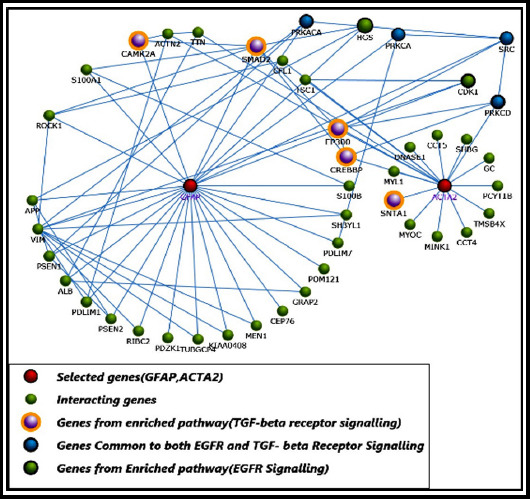
Protein-Protein interaction (PPI) Network of GFAP and ACTA2.

The protein–protein interaction network visualization and its analysis of *GFAP and ACTA2* was performed using FunRich database. The interaction network included the biological pathway enrichment of defined proteins. The PPI network was among differentially regulated interacting proteins of potential retrieved from interaction of *GFAP and ACTA2* in Fig.1. Among selected *GFAP and ACTA2* interacting 44 protein genes, all had interactions with each other as shown in Fig.1. The gene mapping of *GFAP and ACTA2* interacting proteins with their chromosomal location was shown in Table-I. The enzymes represented the major category mapped along with protein kinase C and proto-oncogenes of tyrosine kinase. The leading biological pathways associated with these interacting proteins were signalling by EGFR and TGF-beta receptor signalling as depicted in Table-I.

**Table-I:** Gene Mapping and Biological Pathways Enriched in Interaction of GFAP and ACTA2 shown in Fig.1.

Gene symbol	Protein Name	Chromosome	Map location	Interacting Genes with GFAP and ACTA 2	Biological Pathway	p-value
PRKCD	Protein kinase C, delta	3	3p21.31	CREBBP; PRKCA; EP300; CDK1; PRKACA	Retinoic acid receptors-mediated signaling	p = 0.009
HGS	Hepatocyte growth factor-regulated tyrosine kinase substrate	17	17q25	PRKCD; PRKCA; SRC; VIM; SMAD2;	Alpha6Beta4Integrin	p = 0.01
PRKCA	protein kinase C, alpha	17	17q22-q23.2	PRKCD; PRKCA; SRC; PRKACA; ROCK1	Thromboxane A2 receptor signaling	p = 0.017
RC	SRC proto-oncogene, Non-receptor tyrosine kinase	20	20q12-q13	PRKCD; PRKCA; SRC; CDK1; PRKACA; PSEN1;PSEN2	Signalling by NGF	p = 0.018
CDK1	Cyclin-dependent kinase 1	10	10q21.1	PRKCD; HGS; PRKCA; SRC; CDK1; PRKACA	Signaling by EGFR	p = 0.023
PRKACA	Protein kinase, cAMP-dependent, catalytic, Alpha	19	19p13.1	CREBBP; PRKCD; PRKCA; EP300; SRC; VIM;PRAKCA;APP;SNTA1	TNF receptor signaling pathway	p = 0.043
GFAP	Glial fibrillary acidic protein	17	17q21	CREBBP; PRKCD; PRKCA; EP300; SRC; PRKACA;SMAD2;SNTA1;CAMK2A	TGF-beta receptor signaling	p = 0.051
ACTA2	Actin, alpha 2, smooth muscle, Aorta	10	10q23.3	CREBBP; PRKCD; PRKCA; EP300; SRC; PRKACA;SMAD2;SNTA1;CAMK2A	Regulation of nuclear SMAD2/3 signaling	p = 0.051
CREBBP	CREB binding protein	16	16p13.3	CREBBP; PRKCD; PRKCA; EP300; SRC; PRKACA; SMAD2;SNTA1;CAMK2A	Regulation of cytoplasmic and nuclear SMAD2/3 signaling	p = 0.051
PRKCD	Protein kinase C, Delta	3	3p21.31	CREBBP; PRKCD; PRKCA; EP300; SRC; PRKACA; SMAD2;SNTA1;CAMK2A	ALK1 signaling events	p = 0.076
PRKCA	Protein kinase C, Alpha	17	17q22-q23.2	CREBBP; PRKCD; PRKCA; EP300; SRC; PRKACA; SMAD2;SNTA1;CAMK2A	ALK1 pathway	p = 0.082

The proteins enriched in Signalling by EGFR Pathway were *HGS, CDK1, PRKCA, SRC, PRKCD and PRKACA*. Likewise, Protein-Protein interaction (PPI) Network of GFAP and ACTA2 enriched in TGF-beta receptor Signalling were *PRKACA, PRKCA*, *CAMK2A, SRC, SMAD2, PRKCD, CREBB* and *SNTA1*. It is worth mentioning that *SRC, PRKACA, PRKCA and PRKCD were enriched in both* EGFR and TGF-beta Signalling pathways as shown in Fig.1.

**Fig.2:**
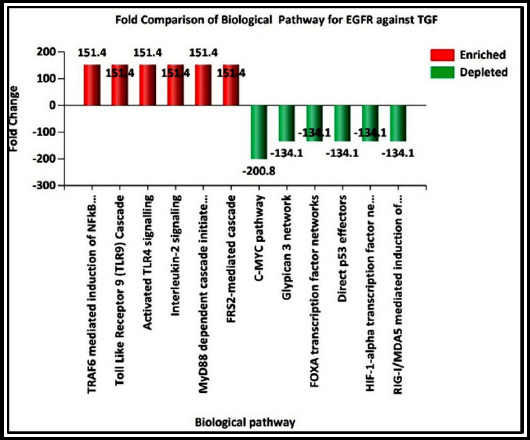
Fold Comparison for Biological Pathway of proteins involved in EGFR Signalling (6) against TGF Beta Receptor.

In liver fibrosis, there were divergent proteome repertoires regarding *EGFR* and *TGF* beta receptor signalling. Superimposed bar chart depicted fold comparison of the differential expression of biological pathway proteins involved in EGFR Signalling (6) against TGF Beta Receptor Signalling (9). The proteins in related to EGFR signalling pathways were enriched up to 150 fold while proteins in *EGFR* signalling were depleted more than 130 fold (Fig.2).

### Differential Expression Genes/Proteins and their Pathways

In Table-II, deep red boxes showed significant enriched pathways were Signaling by EGFR with fold enrichment of more than 2 folds and p-value: 1.51E-05 and TGF-beta receptor signalling with fold enrichment of more than 10 folds and p value: 1.32E-12. The common genes related to these pathways are *PRKCD; PRKCA; SRC; CDK1; PRKACA*, *CREBBP; PRKCD; PRKCA; CAMK2A; EP300; SRC; PRKACA; SMAD2; SNTA1* are shown in the same Table-II.

**Table-II:** Heat Map Showing Differentially Expressed Proteins & their Pathways Interacting with Genes enriched in Signalling by EGFR Pathway and TGF-beta receptor signaling pathway shown in Fig 1.

Biological pathway	Fold enrichment	P-value (Hypergeometric test)	Genes mapped (Signalling by EGFR Pathway)	Biological pathway	Fold enrichment	P-value (Hypergeometric test)	Genes mapped (TGF-beta receptor signaling Pathway)
EGF receptor (ErbB1) signaling pathway	4.894911	1.47E-05	PRKCD; HGS; PRKCA; SRC; CDK1; PRKACA; ACTA2;	Regulation of cytoplasmic and nuclear SMAD2/3 signaling	20.62231	1.32E-12	CREBBP; PRKCD; PRKCA; CAMK2A;
EGFR-dependent Endothelin signaling events	4.891105	1.47E-05	PRKCD; HGS; PRKCA; SRC; CDK1; PRKACA; ACTA2;				EP300; SRC; PRKACA; SMAD2; SNTA1;
Signaling events mediated by Hepatocyte Growth Factor Receptor (c-Met)	4.875939	1.51E-05	PRKCD; HGS; PRKCA; SRC; CDK1; PRKACA; ACTA2;				
Signaling by EGFR	55.02213	8.48E-11	PRKCD; HGS; PRKCA; SRC; CDK1; PRKACA;	TGF-beta receptor signaling	20.62231	1.32E-12	CREBBP; PRKCD; PRKCA; EP300; SRC; PRKACA; SMAD2; SNTA1; CAMK2A;
Signal Transduction	4.464134	0.000291	PRKCD; HGS; PRKCA; SRC; CDK1; PRKACA;	Signaling events mediated by VEGFR1 and VEGFR2	4.864626	6.41E-07	CREBBP; PRKCD; PRKCA; EP300; SRC; PRKACA; SMAD2; SNTA1; CAMK2A;
Signaling by PDGF	57.6263	5.31E-09	PRKCD; PRKCA; SRC; CDK1; PRKACA;	EGFR-dependent Endothelin signaling events	4.891105	6.10E-07	CREBBP; PRKCD; PRKCA; EP300; SRC; PRKACA; SMAD2; SNTA1; CAMK2A;
Signaling by FGFR	47.31531	1.45E-08	PRKCD; PRKCA; SRC; CDK1; PRKACA;	p38 MAPK signaling pathway	25.89165	6.78E-10	CREBBP; PRKCD; PRKCA; EP300; SRC; PRKACA; SNTA1;
TGF-beta receptor signaling	11.79678	0.000169	PRKCD; PRKCA; SRC; PRKACA;	Role of Calcineurin-dependent NFAT signaling in lymphocytes	36.81247	8.49E-08	CREBBP; PRKCD; PRKCA; EP300; PRKACA;

## DISCUSSION

Fibrosis is a characteristic feature of end-stage liver disease and it constitutes a predominant cause of global rise in mortality and morbidity.^1^ Chronic hepatic injury irrespective of cause is characterized by hepatic stellate cells (HSCs) activation, proliferation, and migration within liver tissue.^15^ These HSCs express various mesenchymal markers upon activation^6^. Expression of two such markers *ACTA2* and *GFAP* has been demonstrated in our previous study by using immunohistochemistry.^9^ The management of hepatic fibrosis still remains a challenge therefore the identification of these proteins and their interacting pathways involved is critical in facilitating early diagnosis and designing target therapeutic modalities.^16^,^17^

The Pathway analyses play a vital role understanding biological mechanisms underlying various disease processes. Therefore, they can help in identifying more potent biomarkers using dysregulated pathways.^13^ We used a network-based method to ascertain the dysregulated pathways elaborated in hepatitis C which may build new insights into pathogenesis of liver fibrosis.^18^ *TGF-β/Smad* signaling pathway is known to be one of the key fibrogenic and inflammatory pathways in the liver.^19^ TGF-β1 have been implicated in the process of activating HSCs with the magnitude of fibrosis being in proportion to increase in TGF β levels. Studies have shown that *ACTA2* is associated with *TGF β* pathway that enhances contractile properties of HSCs leading to fibrosis.^20^ The results of our study show that biologic pathways associated with *GFAP* and *ACTA2* were signaled by *TGF β* receptor signaling which is consistent with the previous studies. On the basis of close interaction of proteins, we used PPI networks to identify disease-specific networks. Our study showed a number of proteins enriched in *TGFR* signaling primarily involving *PRKACA, PRKCA, CAMK2A, SRC, SMAD2, PRKCD, CREBB* and *SNTA1*. Moreover, functional enrichment analysis of *GFAP* and *ACTA2* interacting proteins showed 85.7% enrichment of proteins in signaling pathways of *EGFR*. This led to identification of another pathway, the epidermal growth factor receptor (*EGFR or ErbB1*) signaling system, which seems to be strongly associated with the interacting proteins *GFAP* and *ACTA2*. This finding may be due to the facilitation of crosstalk between signaling pathways by *EGFR*, resulting in release of various mediators of inflammation and repair.^11^ The *EGFR* signaling is reported to be a key element in not only fibrosis but also the proliferation of fibrotic liver injury to neoplastic transformation.

The study by Yang et al. has shown that *EGFs* can stimulate proliferation of hepatic stellate cells, which is the primary effector cell, orchestrating the deposition of extracellular matrix (ECM) in fibrotic liver.^16^ *EGFR* showed signaling enrichment of proteins similar to those in *TGFR*, including *PRKACA*, *PRKCA*, *SRC, SMAD2* and *PRKCD*. Protein kinase C (*PKC*) is a group of calcium dependent proteins which regulate embryonic development. Various members of this PKC family have been implicated in progression of cell cycle, apoptosis and differentiation.^21^ Protein kinase A family of proteins is activated in response to G coupled protein receptors^22^ while *PRKCD* plays a key role in autophagy suppression which is achieved by the process of phosphorylation of *AKT* which further activates mTOR, specific for fibrolamellar carcinoma.^23^ In current study, activity of c-SRC decreases with progressive liver fibrogenesis and hepatic stellate cell (HSC) activation. This finding is consistent with literature which reports that inhibition of SRC Kinase promotes HCV replication.^24^ The oncogenic properties of *SRC* family kinases have been reported with various studies upon role of *SRC* as target therapy in the treatment of idiopathic pulmonary fibrosis, systemic sclerosis and glioblastoma. However, its role in liver fibrosis progression is not yet understood.^25^ SRC along with *PRKACA, PRKCA* and *PRKCD* must be further explored to establish their role in target therapy of hepatic fibrosis in chronic hepatitis.

## CONCLUSION

In this analysis, many perilous pathways and genes were identified based on protein-protein interaction of network *GFAP* and *ACTA2*. *EGFR* and *TGF*-beta Receptor Signalling pathways were found to be enriched in liver fibrosis through Protein Interaction studies. *SRC, PRKACA, PRKCA and PRKCD* were enriched and differentially expressed in both *EGFR* and *TGF*-beta Signalling pathways. These signalling pathways and related proteins are the potential targets for new therapeutic agents to combat liver fibrosis resulting from chronic hepatitis C.

### Authors’ Contribution

**ZR** conceived, designed and did manuscript writing along with editing of manuscript.

**SH** did data collection, manuscript writing and editing of manuscript

**HS and SS** did literature review, manuscript writing, statistical analysis and editing

**ZR** takes the responsibility and is accountable for all aspects of the work in ensuring that questions related to the accuracy or integrity of any part of the work are appropriately investigated and resolved.
